# A simple, open and extensible gating Control unit for cardiac and respiratory synchronisation control in small animal MRI and demonstration of its robust performance in steady-state maintained CINE-MRI

**DOI:** 10.1016/j.mri.2021.04.012

**Published:** 2021-09

**Authors:** Stuart Gilchrist, Paul Kinchesh, Veerle Kersemans, John Beech, Danny Allen, Michael Brady, Borivoj Vojnovic, Jurgen Schneider, Jack Miller, Sean Smart

**Affiliations:** a Department of Oncology, University of Oxford, Old Road Campus Research Building, Off Roosevelt Drive, Oxford OX3 7DQ, United Kingdom; b Leeds Institute of Cardiovascular & Metabolic Medicine, University of Leeds, Leeds LS2 9DA, United Kingdom; c Clarendon Laboratory, Department of Physics, Parks Road, Oxford OX1 3PU, United Kingdom; d Department of Physiology Anatomy and Genetics, Sherrington Building, Parks Road, Oxford OX1 3PT, United Kingdom; e Oxford Centre for Clinical Magnetic Resonance Research, Level 0, John Radcliffe Hospital, Oxford OX3 9DU, United Kingdom

**Keywords:** Cardiac imaging, Prospective gating, CINE, Steady state, CAD, Computer Aided Design, cCRT, Conventional cardio-respiratory triggering, CT, x-ray computed tomography, DC, Direct current, DG, Double gating, dTTL_Acquire._, Delay as the respiratory TTL signal instructs the scanner to acquire respiratory gated data and which is used as input for respiratory gating of the cardiac TTL signals, dTTL_ECG_, Delay that instructs the scanner to acquire cardiac gated data, dTTL_NoAcquire._, Delay as the respiratory TTL signal instructs the scanner not to acquire data, dTTL_Threshold_, Delay whilst the respiratory signal exceeds its threshold and generates a TTL signal, ECG, Electrocardiogram, GCU, Gating control unit, LED, Light emitting diode, PET-CT, Positron Emission Tomography and (x-ray) Computerised Tomography, SA, Short axis, PVDF, Polyvinyl difluoride, SPECT-CT, Single Photon Emission Computed Tomography and (x-ray) Computed Tomography, tSSM, True steady state maintenance, TTL, Transistor-transistor logic (actually 0 or 5 V logic control signal)

## Abstract

Prospective cardiac gating during MRI is hampered by electromagnetic induction from the rapidly switched imaging gradients into the ECG detection circuit. This is particularly challenging in small animal MRI, as higher heart rates combined with a smaller myocardial mass render routine ECG detection challenging. We have developed an open-hardware system that enables continuously running MRI scans to be performed in conjunction with cardio-respiratory gating such that the relaxation-weighted steady state magnetisation is maintained throughout the scan. This requires that the R-wave must be detected reliably even in the presence of rapidly switching gradients, and that data previously acquired that were corrupted by respiratory motion re-acquired. The accurately maintained steady-state magnetisation leads to an improvement in image quality and removes alterations in intensity that may otherwise occur throughout the cardiac cycle and impact upon automated image analysis. We describe the hardware required to enable this and demonstrate its application and robust performance using prospectively cardio-respiratory gated CINE imaging that is operated at a single, constant TR. Schematics, technical drawings, component listing and assembly instructions are made publicly available.

## 1 Introduction

Magnetic resonance imaging is the gold-standard clinical imaging modality for the heart, and exquisite control of the scanner is required to minimize the unwanted effects of motion that results from both cardiac and respiratory function. Many other organs of clinical interest are subject to the same cardiac and respiratory motions and inevitably move, often substantially and unpredictably, during normal activity and when at rest. Since MR imaging acquisition times are often long compared to the durations of motion, such motion can substantially degrade the resulting images, typically producing ghosting and other corruptions that are, in general, highly nonlinear functions of the exact motion performed, and the exact MR pulse sequence applied. Due to the volumetric nature of k-space acquisitions common to almost all MRI scans, localised motions can cause significant non-localised image corruptions once k-space has been spatially resolved by Fourier transformation. Small animal cardiac imaging which is the focus of this work is typically performed at high magnetic field, movements well below 1 mm are sufficient to render images non-diagnostic as motion artefacts scale with magnetic field strength [1].

Prospective ECG-based synchronisation of the MR acquisition to the cardiac cycle (‘triggering’ or ‘gating’) [2,3] is widely used to reduce image degradations resulting from motion of the heart, using transducers such as those used for pulse oximetry, or ECG-detection electrodes placed on (or in) the subject. In comparison to peripheral pulse-oximetry gating, ECG gating is more precise, less variable, and preferable for advanced cardiac imaging [4]. However, ECG has a number of fundamental limitations, largely as a result of electromagnetic induction in the ECG detection circuit. The main sources of induction are: motion of the circuit through the magnetic field [5]; the magnetohydrodynamic effect [6]; and direct pickup from the rapidly switched magnetic field gradients [6]. Though all of these corrupt the ECG signal, it is the latter that most often corrupts it beyond use in small animal imaging. A simple approach to avoid the problems caused by rapidly switched magnetic field gradients is to precede each R-wave with periods during which the gradients are not applied so that the nuisance signal can decay prior to occurrence of the next R-wave. This necessarily assumes a near-constant R-R interval and precludes the capture of the entire cardiac cycle, although the delay typically required is small. Data acquisition, and typically scanning, is suspended after the completion of one imaging block until the next R-wave arises. The second motion-derived disturbance arises from respiration, which is usually avoided by suspending imaging during the breath motion. This is the motion control scheme used in conventional cardio-respiratory triggered (cCRT) CINE imaging [7,8]. However, such scans operate with a mix of repetition times TR: the short TR of the CINE block; and the ‘TR’ invoked whilst awaiting the next R-wave trigger signal. The duration of the latter ‘TR’ may range from a few to several hundred milliseconds while a breath occurs and even in the optimum case where a healthy heart is imaged, it is known that the R-R interval itself is physiologically variable [9,10]. As a direct consequence of the unpredictable delays the resulting magnetisation amplitudes will vary between R-R intervals and even more so between respiratory events, which in turn will result in ghosting. Low flip angles, with their intrinsically compromised signal and contrast/noise performance, are then required in order to minimize T_1_-derived amplitude modulations. The so-called ‘double gating’ (DG) techniques [[11], [12], [13]] minimize amplitude modulation by delivering RF pulses following each heartbeat, even during the breath; but the resulting intensities are inevitably subject to the hiatus in-between the end of one CINE block and the start of the next. As a result, the intensities are not quantitative. This is once again a consequence of a mixed-TR operation, and scans remain prone to further corruption in the event of missed triggers and unstable heart rates. To stabilize intensities, steady state maintained, constant TR methods would be advantageous; but these require gradients to be applied throughout the scan and this is widely reported to corrupt the ECG trace [14,15]. A number of real-time signal processing methods have been reported for R-wave detection in the presence of gradient-induced noise [15] and the magnetohydrodynamic effects [16]; but they require a non-negligible time to operate and typically invoke delays of up to ca. 30 ms following the R-wave, which is a significant fraction of the R-R interval of the rat or mouse. Even widely used commercial systems which are still prone to gradient-induced corruptions of the ECG feature delays exceeding 10 ms. Whilst this may be adequate in some cases, for example structural imaging, where only a reproducible temporal alignment with the heartbeat is required, it may be inadequate in other cases, such as multi-frame CINE MRI estimations of cardiac function, where imaging immediately following the R-wave is required. These problems can be overcome through the use of retrospective gating techniques [12,18,19] which often use the MR signal itself for data driven cardiac synchronisation. In such methods, each k-space datum is repeatedly acquired over at least one complete cardiac cycle and the data are reordered after the scan is completed to produce a CINE-resolved image. In cases where the resolution of the complete cardiac cycle is not required, the retrospective gating techniques impose a significant time penalty as only one k-space line per cardiac cycle is acquired. We have recently demonstrated that prospective gating using R-wave based gating control can enable a substantial, >64-fold, acceleration in imaging speed compared to that which could be achieved retrospectively [20]. This is achieved by acquiring data using a k-space ordered segmentation scheme in which multiple k-space lines are acquired, in a pre-defined and controlled manner, after each R-wave is detected. At all times the steady state is maintained by application of RF and gradient pulsing at a constant and short TR whilst ECG gating signals are robustly generated; a scan mode termed ‘true steady state maintenance’ (tSSM). While this is not a complex scan mode, it depends fundamentally on the availability of a real-time signal processing apparatus to generate control signals robustly and with the minimum possible latency even when significant signal corruptions are expected. In order to link the operation of this scan mode with occurrence of the R-waves, we have developed a Gating Control Unit (GCU) device that robustly produces gating control signals which are interpreted in real time by the scanner such that robust R-wave synchronisation is achieved with concurrent, automatic and on-the-fly re-acquisition of data that are corrupted by respiratory motion.

In this paper, we describe and demonstrate operation of the GCU. The supplemental information provided with the paper includes assembly instructions so that others wishing to replicate this work can do so, with code and other supporting computer files available. The device created is open-source, open-hardware, but at present requires a commercially available analogue-to-digital converter and data acquisition/display device and user interface.

Additionally, the quality of scan control afforded by the GCU is validated with high quality detection of the R-wave, even in the presence of a continual stream of strong and rapidly switched imaging gradients as used in constant TR, cardio-respiratory synchronized CINE imaging in the mouse heart. High quality CINE image series that are isointense over the cardiac cycle, regardless of the degree of T_1_-weighting used, are produced, and high-throughput operation in vivo is demonstrated. It is hoped that this affordable, open, and flexible system will therefore be of benefit to the scientific community.

## 2 Methods

### 2.1 MRI system

MRI was performed at 7.0 T preclinical MRI scanner (Varian VNMRS) using a 26 mm (internal diameter), 35 mm (long) quadrature-driven birdcage coil and a gradient set with an inner bore diameter of 120 mm and operating at G_max_ = 400 mT/m with a switching time of 160 μs to maximum (2500 mT/m/ms slew rate).

### 2.2 MRI pulse sequence

**Fig. 1:**
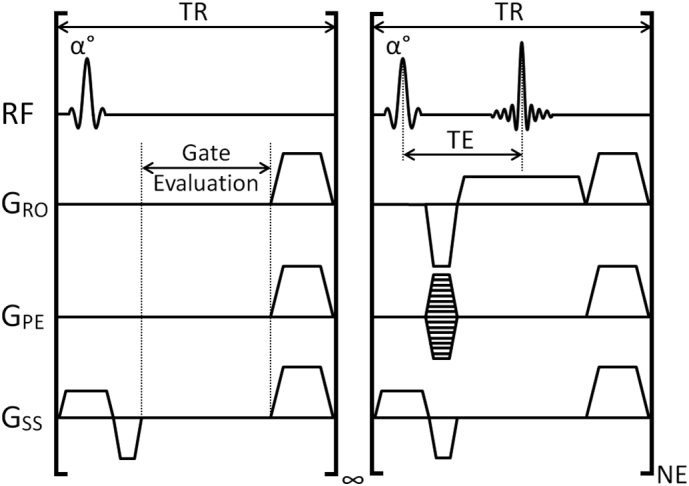
Pulse sequence looping structure. The infinite loop of the ‘Gate Evaluation’ TR element is terminated when the gating logic control signal defines the occurrence of a R-wave. This invokes entry into the CINE loop which consist of NE executions of a single k-space line acquisition constitute a CINE block. When the number of executions of the Gate Evaluation loop exceeds a specified threshold a breath is declared, and the 2 preceding CINE blocks are re-acquired at the end of the same breath.

A 2D CINE FLASH scan using true steady state maintenance, cardio-respiratory gating and dynamic re-acquisition controls as shown in Fig. 1. This scan mode and the GCU as developed have been used successfully in thousands of scans while under development in our institution, including in conjunction with 3D gradient echo and 3D balanced-SSFP imaging.

### 2.3 MRI parameters

The CINE sequence described above was performed with RF and gradient spoiling and used 1 mm thick slices excited with 1 ms duration Gaussian shaped RF pulses. A receiver bandwidth of 89 kHz was used with TE = 1.89 ms and a constant TR = 4 ms. A 128 × 128 image matrix covered a FOV of 27 × 27 mm. 20 CINE frames (over a time of 80 ms, thus enveloping the contraction of the heart) were acquired with a count of >25 steady state loop operations (100 ms) indicating that a breath had occurred (or that an R-wave had been missed), invoking re-acquisition of the two preceding CINE blocks. This is a rather stringent setting for invoking re-acquisition, and the count of steady state loop operations can be increased such that missed R-waves do not instruct for re-acquisition, but breaths do. This scan was repeated with the cardio-respiratory synchronisation controls modified to effect cCRT and DG control schemes, the latter operated without automatic re-acquisition but with manual control of the respiratory interval imaging period. The dependence of the mode of cardio-respiratory synchronisation and flip angle (FA) upon image presentation, and the dependence of ECG trace quality upon scan orientation were examined as summarised in Table 1.

**Table 1:** Definition of scan parameters used for assessment of image and ECG quality.

Scan Mode	FA (°)	Orientation	No’ repeats	Scan ID
cCRT	5	Short axis	10	ID1
cCRT	10	Short axis	10	ID2
cCRT	15	Short axis	10	ID3
cCRT	30	Short axis	10	ID4
DG	5	Short axis	10	ID5
DG	10	Short axis	10	ID6
DG	15	Short axis	10	ID7
DG	30	Short axis	10	ID8
tSSM	5	Short axis	10	ID9
tSSM	10	Short axis	10	ID10
tSSM	15	Short axis	10	ID11
tSSM	30	Short axis	10	ID12
tSSM	15	Sagittal	1	ID13
tSSM	15	Coronal	1	ID14
tSSM	15	Axial	1	ID15

Synchronisation modes are given as ‘cCRT’ (conventional cardio-respiratory triggering), ‘DG’ (double gating) and ‘tSSM’ (true steady state-maintained gating). Note that short axis (SA) views use gradient strengths that can be up to √3 stronger than those required for the axial, sagittal and coronal views with a commensurate increase in ∂B/∂t and interference in the ECG signal.

In 2 further mice, each scanned 3 times during a single anaesthetic session, the same tSSM CINE scan was operated in SA view with 10 contiguous 1 mm short axis slices covering base to apex so as to assess robustness of the technique under routine scan operation conditions. Slice positioning was performed using the same sequence which was features an option ‘localiser’ scan mode in which a segmented acquisition with 4 phase encode steps acquired in a centre-out order per R-wave (the number of steps and ordering were parameterised and can be set freely by the user), and which featured an interactive slice positioning tool that placed the next slice to be imaged orthogonally to the currently displayed slice such that slice positioning and acquisition of each localiser image typically took ca. 20 s.

Pulse sequences and associated image reconstruction support files are available for download, see Research Data.

### 2.4 Animal model

For the examples presented in this paper, three mice (25–30 g, male, B6SJLCD45-WT mice, bred locally) which were housed in individually ventilated cages prior to imaging were used. One mouse was used to generate the data for presentation of the ECG signals and validation images. Two mice were scanned in ‘high-throughput mode’ such that each animal was positioned in the scanner for no more than 10 min at a time before being removed with the other animal then positioned in the scanner. Each subject loading was followed by manual global shimming, the acquisition of a series of localizer images to define the short axis of the left ventricle scan view, and the acquisition of 10 sequentially acquired CINE images in short axis view. The CINE images were reviewed for basic image quality before the animal was removed from the scanner. This rapid demonstration of high-quality, gold-standard cardiac images is only possible with exceptionally robust ECG gating.

Anaesthesia was induced and maintained using isoflurane (1–3%) in an 30% oxygen-enriched air maintaining respiration in the range 40–60 breaths/min. Rectal temperature was monitored using a fibre optic based probe and signal conditioner unit (ACS-P4-N-62SC and OTP-M, Opsens Inc., Quebec, Canada), and maintained at ca. 37 °C using a homeothermically controlled copper resistor element [21]. The probe tip was coated in epoxy glue for a smooth, rounded tip and lubricated with ultrasound imaging contact gel (Aquasonic 100, Parker Laboratories Inc., USA) for insertion.

Respiration was monitored using a novel MR and CT-compatible piezoelectric device, based upon that previously described [22] that consisted of a strip of piezoelectric polymer (110 μm thick unipoled polyvinyl difluoride (PVDF) sheet, 180 × 180 mm, Precision Acoustics, UK) that was painted with a stripe of conductive particulate graphene paint (Elicarb, Thomas Swan, UK) on each side. The conductive path for exteriorisation of the piezoelectric current was formed by placing a pair of CT-compatible ECG electrodes (Neotech Micro, Neotech, USA), one on each side of the painted PVDF strip. The insulated carbon electrode wires were cut to 50 mm in length and formed into a twisted pair and coupled to a 2-core shielded cable (749–2544, RS Pro., UK) using a 2.5 mm pitch RCY series connector system (Japanese Solderless Terminals) which passed through an RF filter (SCI 56–725-001, Spectrum Control Inc., USA) to the respiration signal amplifier (see 2.5).

Animals were prepared for ECG by shaving an area of approximately 2 cm^2^ centred on the sternum using an electric shaver (Wella Contura, Wella, USA), and by depilating this area further with hair removal cream (Veet, Reckitt Benckiser). An ECG detection system consisting of a pair of commercial CT-compatible ECG electrode pads (Neotech Micro, Neotech, USA) that embedded into the animal positioning cradle. At the start of the imaging session the surface of each pad was moistened with a drop of water, and for each subject loading the shaved chest of each mouse was moistened with ultrasound imaging contact gel (Aquasonic 100, Parker Laboratories, Inc’., USA), and then positioned on the pads. No further manipulation of the animal was necessary. The electrode wires were cut to 100 mm in length, formed into a twisted pair and terminated in a RCY connector for coupling to a 2-core shielded cable (749–2544, RS Pro., UK) that was passed through an RF filter (SCI 56–725-001, Spectrum Control Inc., USA) and on to the ECG signal amplifier (see 2.5).

Animals were recovered from anaesthesia without incident and returned to their home cages at the end of the procedures.

### 2.5 Physiological monitoring and recording

Respiration and ECG signals were amplified (Biopac DA100C and MRIECG100 amplifiers, respectively), digitized, displayed and recorded using a conventional physiological recording device (Biopac MP150 with interface unit UIM100C, Biopac Inc., Goleta, USA). The MRIECG100 amplifier has a slew-rate limiter in order to reduce gradient interference, and the standard built-in audio-filters invoke a 4 ms signal propagation delay. The DA100C amplifier has a 10 Hz low pass filter which invokes a delay >50 ms to the respiration signal and which is accounted for with the dynamic re-acquisition. The ECG and respiration signals were gain-adjusted on a per animal basis, as required, to fit within the ±10 V range as required by the MP150 digitiser and were also passed to the custom-made Gating Control Unit (GCU). A commercially available ECG preamplifier and data recording/acquisition system was chosen to reduce the burden of medical device certification and isolation, extensive GUI software programming, and much design effort needing to be spent on comparatively commodity hardware.

### 2.6 Gating Control Unit (GCU)

The GCU was controlled by three individually programmable microprocessors (Arduino Uno), each interfaced with a series of variable resistor dials, 2-way switches, LED indicators, I/O ports and power input. See Research Data for explicit details including component part numbers, pinout label descriptions, assembly instructions, microprocessor programs and software installation instructions).

As the input voltage range for the microprocessors was 0 to +5 V the amplified respiration and ECG signal were conditioned from ±10 V to 0 to +5 V using a custom-made voltage scaling board (see Research Data for description, circuit diagram and component part numbers). The GCU incorporates gain controls to allow the user to manage the signal within the 0–5 V range.

**Fig. 2:**
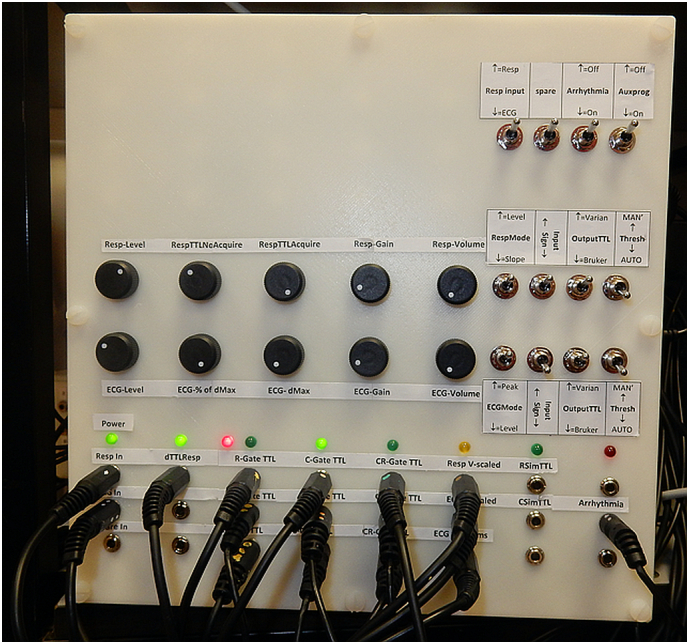
The Gating Control Unit (GCU). A full description of the port and control switch and dial labelling and functionality is provided in Research Data. Unlabelled ports and switches are currently unused and available for future expansion. The respiratory, cardiac and cardio-respiratory gating outputs are triplicated such that signals can be submitted to: the physiological recording system; conventional trigger inputs on the scanner; and real time gating control inputs on the scanner, all without the need for external cable splitters.

The GCU was fabricated in a casing produced using a 3D plastic printer (Stratasys F170) from technical drawings made in CAD software (Solidworks 2018–2020, Dassault Systems) The GCU assembly is shown in Fig. 2, again with detailed descriptions given in Research Data.

The MRI scanners used were configured such that 0 or 5 V logic control outputs from the GCU are declared as being instructions either to acquire, or not to acquire imaging data (or vice versa), and the scanner interprets these signals such that the imaging or steady-state maintenance loops are appropriately operated as instructed by the processed signals. The sense of these signals can be switched by the operator in accordance with the requirement of the scanner to be controlled (some scanners use 0 V as the instruction-to-image, others use 5 V).

### 2.7 Respiration signal conditioning

**Fig. 3:**
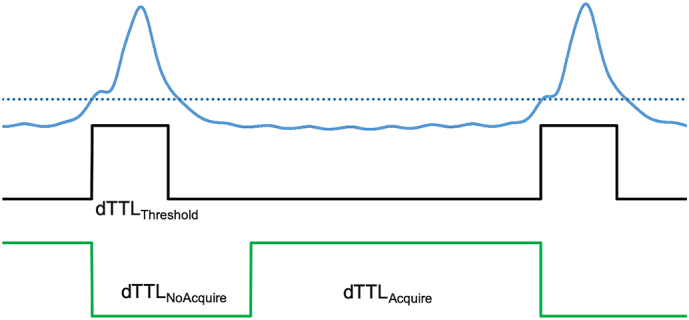
Analog-to-digital conversion of the respiratory signal and the generation of the TTL control signal that instructs the scanner either to acquire, or not to acquire MRI data. dTTL_Threshold_ is the duration of the control signal formed in response to crossing the threshold (indicated with the blue dotted line). This signal is terminated when the signal crosses the level defined as being +20% of the difference between the threshold onset and maximum voltages. This is because asymmetries in the breath profile sometimes resulted in artificial, apparently long breaths in cases where the baseline was not reached in-between breaths. As the breath is still in progress as the threshold is crossed at the trailing edge of the breath, a user-variable potentiometer allows the generation of the extended control signal which lasts, in total, for a duration dTTL_NoAcquire_ and is set such that its duration exceeds that of the actual breath. At the end of dTTL_NoAcquire_ period the TTL signal is raised to 5 V for user-variable duration dTTL_Acquire_ such that if the next breath occurs during this period the signal is terminated and the next dTTL_NoAcquire_ period begins. (For interpretation of the references to colour in this figure legend, the reader is referred to the web version of this article.)

The analogue respiration signal gain was adjusted to ensure it was within the 0–5 V range and was digitized every 10 ms. A simple 2-point differential computed from adjacent data points was calculated in order to act as a high pass filter and remove low frequency baseline drifts. A threshold based binarisation of the filtered signal performed as shown in Fig. 3, gives a TTL pulse of duration, dTTL_Threshold_. The threshold is manually adjusted on a per subject basis, as necessary, to ensure robust detection of each breath. The breath was in progress as both the leading and trailing thresholds were crossed so the trailing edge was extended by a user-variable duration in order to ensure that the binarised respiration signal encompasses the trailing end of the breath, and with a total duration dTTL_NoAcquire_ This is followed by the ‘image acquisition’ period (dTTL_Acquire_) which was maintained until the next breath was detected unless manually shortened under GCU control (emulating the modus operandi that is most often employed in order to avoid data corruptions resulting from motions occurring at the onset of the next breath). In the presence of multipolar respiration signals the dTTL_NoAcquire_ period should extend beyond the end of the multipolar signal activity to minimize motion across the whole body. Note that respiration generates motion over the whole body that is continuous, and which propagates such that the body is never actually static, even during the inter-breath period.

As the breath cannot be detected until it is in progress, the CINE sequence itself automatically invokes re-acquisition of the n-data blocks that were acquired immediately before the breath was detected at the next available opportunity once the same breath is completed once the next dTTL_Acquire_ has commenced.

The use of commodity and easily programmable hardware enables the rapid development of subsequent additions to this device. As a simple proof-of-principle of real-time signal conditioning improving ECG detection, the GCU also provides a user with the option to use the gradient of the slope of the leading edge of the analogue respiration signal as the input that generates the start of dTTL_Threshold_. Again, this delay is terminated as the signal returns through the voltage corresponding to 20% of the voltage excursion between detection and peak.

### 2.8 ECG signal conditioning

**Fig. 4:**
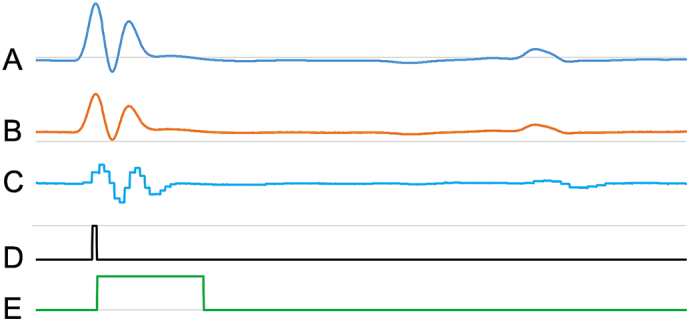
Analog to-digital conversion of the ECG signal and the generation of the gating control signal. A) shows the post amplification ECG acquired during operation of the CINE scan described later. The width of the R-wave complex was ca. 7 ms after it had passed through the filters in the amplifier; its true width, based upon the broadening of test signals passed through the same signal pathway was ca. 2–3 ms, and its peak was delayed by 4 ms. B) The post amplification and voltage scaled ECG signal. C) representation of the GCU-digitized signal with the 4 ms differential that shows the step that initiates generation of (D) as the sampling for peak detection switches to trace B. D) represents a marker signal showing when the signal measurement switches from the baseline-flattened differential to sampling the signal from Trace B, for detection of the signal's peak in a manner that is independent of the signal amplitude or DC level [note that Traces C and D are not displayed in actual use]. E) shows the control signal that is submitted to the scanner; its duration, dTTL_ECG_ is user variable and its duration should exceed one TR with a duration that is shorter than the imaging, in this case, CINE block.

The raw, scaled analogue ECG signal was gain-adjusted to ensure it fitted within the 0–5 V range and was initially digitized at 1000 samples/s. A 2-point difference was calculated from data points acquired 4 ms apart, in order to act as a high pass filter and remove low frequency baseline drifts. The choice of 4 ms interval was determined empirically to give good performance. Once the R-wave onset was detected, using a threshold setting on the rapidly changing differential signal, the voltage-scaled ECG signal was digitized at 10000 samples/s, and the turning point, indicating the R-wave peak was defined. This threshold was manually adjusted on a per subject basis as necessary and allowed detection of R-waves during the breaths. The ECG gating control signal was generated immediately after the R-wave peak was detected, with the duration of this signal (dTTL_ECG_) being set under operator control. At the same time the sampling reverts to the voltage-scaled ECG signal every 100 μs, as shown in Fig. 4. An additional combined cardio-respiratory gating control signal is output from the GCU such that only those ECG gating control signals produced whilst the respiratory gating signal is in the dTTL_Acquire_ period.

## 3 Results

**Fig. 5:**
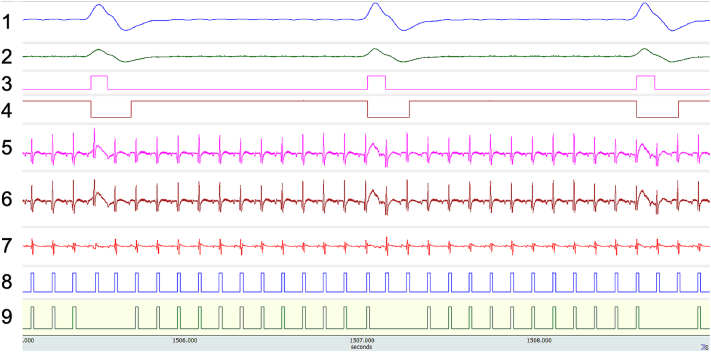
The complete signal pathways for generation of respiratory, cardiac and cardio-respiratory gating control signals acquired during the acquisition of a short axis CINE scan for scan ID11.

The suite of tools we have developed allows cardiac synchronisation to be performed in conjunction with imaging in the steady state and in a manner that is simple-to-use, compatible with high throughput operation and non-invasive to the animal. Fig. 5 shows the complete respiratory and ECG signal pathway as acquired during a short axis CINE scan and is representative of the general case; Table 2 summarises the content of each trace.

**Table 2:** Channel and signal description for Fig. 5.

Trace	Function
1	Raw analogue respiration signal.
2	Voltage-scaled respiration signal.
3	Threshold-based respiration TTL of duration dTTL_Threshold._
4	Respiration gating control TTL. dTTL_Acquire_ and dTTL_NoAcquire_ are the periods during which data are or are not collected respectively. This control mode is dynamically adaptive to the instantaneous breathing rate and allows the maximally efficient, motion desensitised imaging to be performed.
5	Raw ECG signal.
6	Voltage scaled ECG signal.
7	Differential ECG signal. Note the respiration-induced voltage is largely eliminated.
8	Cardiac gating TTL signal.
9	Cardio-respiratory gating control signal: Boolean ‘AND’ combination of Traces 4 and 8.

**Fig. 6:**
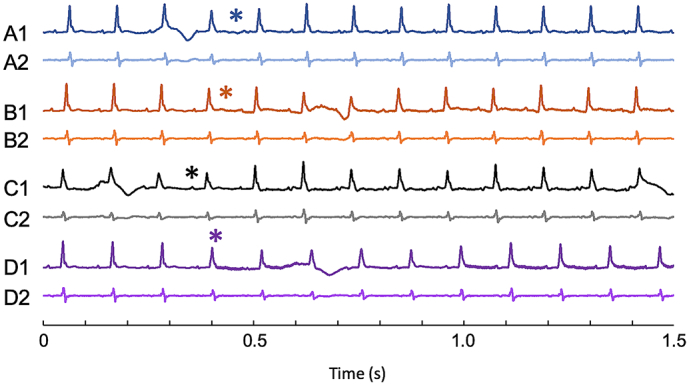
Shows the ECG traces acquired over the few seconds covering the start of CINE scans operated with: sagittal (A1, A2: ID13); coronal (B1, B2: ID14); axial (C1, C2: ID15); and short axis (D1, D2: ID11) orientations. Dark ECG traces (suffixed 1) show the voltage-scaled ECG signal, and the lowlighted traces (suffixed 2) show the differential ECG signals with the removal of the respiration induced voltages. Dots indicate when each scan started. Gradient-induced noise was visible on all traces (but small enough to be largely obscured by the line thickness of the plots) and every R-wave generated a gating control signal as shown earlier in Fig. 5.

Fig. 6 shows ECG traces for scans acquired in sagittal, coronal, axial and short axis views, the latter requiring gradients up to √3 time stronger than the others. Note that every inter-breath R-wave generated a gating signal, and that gradient-induced noise was low. In this case all of the intra-breath R-waves generated gating signals; this is usually, but not always so and where it is not this is a result of excessively large respiration signals which can be avoided with repositioning. We have observed that the level of gradient noise is variable, sometimes reaching approximately 20% of the R-wave peak-to-peak voltage and that this is related to sample positioning. However, when using this system it has always been possible to acquire ECGs of sufficient quality to acquire cardio-respiratory gated scans for imaging in the steady state. Control signals for respiratory-only, cardiac-only and cardio-respiratory gating are generated throughout and the duration of the dTTL_NoAcquire_, dTTL_Acquire_ and dTTL_ECG_ are all user-controlled variables.

**Fig. 7:**
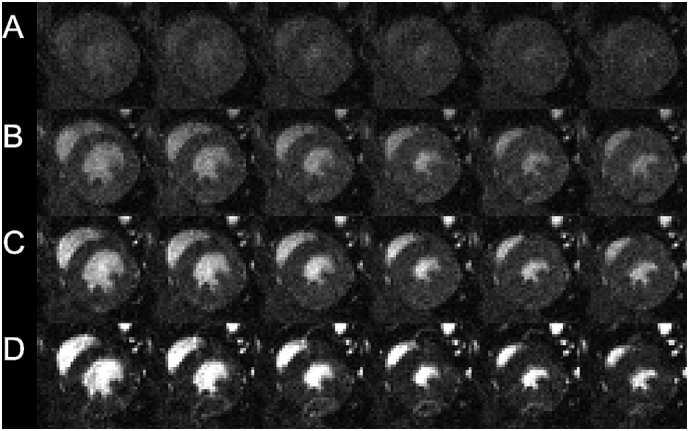
Shows the odd-numbered CINE frames from diastole through to systole for short-axis view scans operated with A) FA = 5 (ID9), B) FA = 10 (ID10) FA = 15 (ID11) and D) FA = 30 (ID12). Note the consistency of intensity along each row afforded by maintenance of the T_1_-weighted steady state throughout the CINE series. Ghosting, consequent to turbulent flow at the approaches to end systole and end diastole are unavoidably present and increase in conspicuity as the flip angle is increased and the inflowing blood signal becomes dominant (note the arc of intensity in the lower half of the heart muscle for Row D, FA30).

**Fig. 8:**
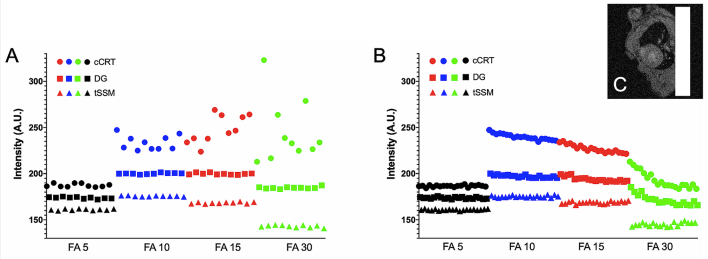
Signal intensity stability for A) the first CINE frame over a 10-repeat dynamic series (long term) and B) over the CINE frame time dimension (short term) for the ROI highlighted in (C). Points in black, blue, red and green represent use of 5, 10, 15 and 30 degree flip angles, respectively, whilst circle, square and triangle icons represent cCRT, DG and tSSM gating scan modes. At FA = 5 all methods provide good long and short term stability but the contrast of blood and muscle is poor (see Fig. 7). At FA ≥ 10 both DG and tSSM offer good long term stability (see A) whilst tSSM offers increased stability within the CINE frame dimension (see B). At FA30 incoherent blood flow results in ghosting for all gating methods so whilst signal stability is good in the muscle ROI selected, blood-derived ghosting is present across the whole phase encode field of view that is in line with the heart (see Research data for complete image files). (For interpretation of the references to colour in this figure legend, the reader is referred to the web version of this article.)

Short axis view CINE imaging was successfully performed in the steady state as shown for one slice through the heart. Fig. 7 shows alternate CINE frames running from end diastole to end systole from tSSM scans acquired with FA 5, 10, 15 and 30°, respectively, with a scan time of approximately 25 s per slice. As expected, increased contrast of the inflowing blood and the myocardium was found at the higher flip angles, as were increased levels of residual inflow-related ghosting. Image intensities and instabilities (reflecting the instantaneous amplitude of the longitudinal magnetisation) were systematically modulated as a function of method of synchronisation control and diminished in the order cCRT >DG > tSSM. Excluding the residual ghosting that arises from inconsistent blood flow in and around the heart and lung, the tSSM longitudinal magnetization amplitude (and therefore image intensity) depends only upon M_0_, T_1_, T_2_*, FA and TR; DG scans are additionally modulated by the R-R interval which may be erratic and by unpredictable intervals resulting from missed R-wave detection; cCRT is further modulated by erratic inter-breath intervals. It is these additional modulations that increase the cCRT and DG signal intensities and instabilities compared to tSSM. Fig. 8 summarises the image amplitude modulations for the ROI positioned in an area of the body, which not prone to any ghosting derived from residual cardiac instability so presenting a faithful representation of the cardio-respiratory gating control. Complete image volumes are available in Research Data.

To demonstrate high throughput capability of the apparatus, 2 mice were scanned alternately, three times each in a total of under 50 min, with new slice positioning defined for each repeated scan. Table 3 summarises the animal loading schedule.

**Table 3:** Timings of the animal changeovers for the three repeated scans of mice numbered C4 and C3.

Animal ID	Repeat	CINE scan start-time (minutes)	Scan ID
C4	1	0	ID16
C3	1	+10	ID17
C4	2	+11	ID18
C3	2	+9	ID19
C4	3	+8	ID20
C3	3	+8	ID21

The CINE scan start time is referenced to the previous scan start time. The last image; mouse C3: repeat 3 (ID21) took 4 min to acquire so the total scan time from the start of the first to the end of the last CINE scan was 50 min. Note that this does not include the set-up time for the first scan as, in our experience, the set-up of the first animal in any session takes longer than for the subsequent animals. Regardless, the entire 10-slice CINE imaging procedure for 6 mice, from induction of anaesthesia in the first mouse to the removal of the last mouse from the scanner, can be comfortably accommodated in under one hour.

**Fig. 9:**
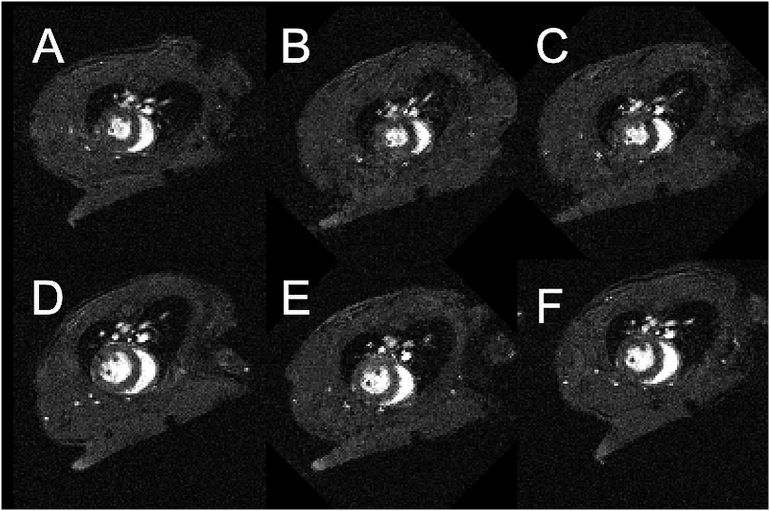
The first CINE frame from the fifth slice above the base for the three repeated scans for two mice. A:ID16, B:ID18, C:ID20, D:ID17, E:ID19, F:ID21. All images are from 10 slice, 128 phase encode, 20 CINE frame image volumes and were acquired within a single 50 min period. Note the quality of slice (re)positioning and image contrast. Also note that, due to an unresolved feature of the legacy scanner software used in this work (VnmrJ 4.2 with custom patches, see Research Data), the FOV in images B, C and E of these images is rotated by 45 degrees relative to the A, B and F.

Fig. 9 shows the fifth slice from the base of the heart for each of the 3 loadings of each of the 2 mice. Multislice CINE imaging data, along with records of the physiological traces, are available for inspection in Research Data.

## 4 Discussion

High quality ECG measurement has been demonstrated in the presence of strong and rapidly switched imaging gradients. Though the ECG traces shown in Fig. 5, Fig. 6 were acquired with a high gradient demand considered normal in small animal MRI, ECG signal corruption did not prevent robust generation of scanner control signals in close (<4 ms) synchrony with the actual R-wave event. The surface-detection ECG pads do show an increased level of gradient noise compared to the invasive platinum needles we have previously described [16,19] which we believe is primarily a result of the increased induction loop area of the pads. When the same ECG signal carrier cable is used with the platinum electrodes described previously [16,19] the gradient noise is barely detectable so suggesting that if an ECG signal is idiopathically poor that needles may be preferable to the surface pads. Further work in order to characterise the gradient noise performance of different ECG pad size, spatial positioning and the use of higher strength gradient sets is warranted. In the general case, however, the use on non-invasive ECG detection using pads may be considered preferable on grounds of animal welfare as well as ease-of-use; the clean ECG is produced within seconds of starting the positioning the chest on the pads. Regardless of the means of ECG detection, these CINE-MRI scans could be operated in the steady state with data either acquired or not acquired according to the state of the faithfully generated cardio-respiratory gating control signals, and this was shown to be effective regardless of the gradient orientation with respect to the ECG detectors. It was also shown that the experimental set up was very easy-to-use, indeed an average animal changeover time of 9.2 min/scan (*n* = 5 changes) was readily achieved; effecting a potential throughput >6 mice/h, approximately 50 mice/day.

Furthermore, as it was shown that the image intensities when using tSSM are stable over a range of flip angles (actually T_1_-weightings) whereas they cannot be when using cCRT or DG synchronisation. For tSSM the magnitude of the magnetisation is entirely predictable and is produced in a way that is insensitive to changes in respiration rate, R-R interval and missed heartbeats. The prospective gating control method used also offers the potential for reduced minimum scan time compared to the retrospective gating methods which offer the same image fidelity advantages afforded through the use of constant TR and stable magnetisation amplitudes. Retrospective gating requires a repeated acquisition of phase encode (projection) data that is performed over at least one complete cycle of the motion to be controlled, and it is common to acquire up to and beyond ten repeats of each cycle in order to complete a scan. Whilst this does provide a commensurate increase in SNR through signal averaging it comes at the cost of increased scan time which may preclude the examination of rapid dynamic processes such as gadolinium contrast agent uptake, that are accessible to prospective gating scans [16]. Where SNR is adequate prospective gating allows minimised scan times because CINE data that are not corrupted by respiratory motion, from end diastole to beyond end systole are acquired just once per phase encode (projection) step. In cases where SNR is inadequate with a single average measurement then the scan can simply be repeated. Further investigations into the relative performance of this prospective gating approach to CINE imaging with existing methods was beyond the scope of this work but a quantitative assessment of the relative accuracy, precision and speed-of-imaging would be of value.

We have previously demonstrated applications of prospective gain in whole body DCE-MRI [20] and T_1_ mapping [23] using Variable Flip Angle 3D-FLASH imaging, and lung tumour imaging [24] using 3D-bSSFP with this gating control system for cardio-respiratory synchronisation. Respiratory-only gating has also been previously demonstrated using bSSFP [25] and FSE [26,27] and it is noted that both cardio-respiratory and respiratory-only gating schemes are applicable to other types of both 2D and 3D scans beyond the heart. All were enabled using the GCU as described.

The ECG signal processing pathway, as described, features a 4 ms signal propagation delay. This delay can be reduced to <1.5 ms by changing resistors in the commercially available amplifier unit's audio-filters (see Research Data for further information) though robust performance at this short latency currently requires the use of invasive needle ECG detectors (data not presented). This gives a much-improved synchronisation with the R-wave compared to those described previously [16,17] for which delays exceeding 20 ms were reported. As the time delay between end diastole and systole is ca. 40 ms [28] such long time delays are inappropriate for use in CINE imaging. When using the short latency hardware, for this prospectively gated steady-state maintained CINE scan, the residual asynchrony of imaging the first datum following R-wave will be dominated by the asynchrony between the instantaneous occurrence of the R-wave and its position within the TR period (4 ms for the high-throughput CINE scans presented) during which the CR-gating signal is detected, the first imaging datum being acquired during the next TR period. Necessarily, the first CINE frame is acquired with a linearly distributed jitter of 1 TR period centred at +0.5*TR post R-wave detection. Short TR is therefore advantageous, and an evaluation of the state of the TTL control signal that is faster than, and asynchronous to, TR would be more so.

The GCU used to generate the gating control signals is simple to build; it is mounted within a 3D printed casing and, beyond production of the voltage scaling board, which is included as to maximize sensitivity and robustness in general usage, can be assembled without specific electronics expertise. This voltage scaling board can be produced by commercial supplier at low cost (see Research Data] with only insertion of through-hole mounted DC-DC converter, potentiometer and headers for the developer to perform. The penalties for omission of this board are that a 4-fold reduction in dynamic range is incurred as the input signals for the GCU must, otherwise, lie in the range 0–5 V, rather than ±10 V specified for the MP150 recording unit, and manual gain and DC offset adjustments that may be non-intuitive are required. A microprocessor that can digitize in the range ± 10 V would circumvent the need for this.

The GCU has been deployed successfully on MRI scanners manufactured by both Varian and Bruker, on a SPECT-CT scanner manufactured by MILabs and on a PET-CT scanner manufactured by Siemens. So long as a scanner's gating capabilities can be controlled using TTL signals then this GCU can be made compatible with said device. Furthermore, the GCU is a user-programmable device, based around a microprocessor that is marketed for the hobbyist, with currently unused I/O ports and switches. Reallocation of the existing ports, dials and switches is also straightforward. As such, new functionality can be introduced with ease and the system becomes extensible. Since its first incarnation the system has been developed to include audible alerts to indicate when the breaths and R-waves have been detected using the dTTL_Acquire_ and dTTL_ECG_ periods to control the sound, and further enhancements such as automated gain control and generation of arrythmia rejection control signals are in development. Simulated respiration and cardiac signal generation has also been implemented, each having a slightly variable period (mean, (span)) of 1000(±100) and 134(±10) ms respectively in order to simulate real physiological signals such that live animals are not required for scanner system development, quality control testing, and similar.

The architecture of the GCU allows the use of other microprocessors and other physiological signal detection units, and the assembly of such a GCU could follow the design principles as described in this report. We note that the ease of programming Arduino units, and the extant links this GCU system provides between them and the scanner console and acquisition computer, means that future work could utilise this platform as an easily extensive real-time analysis suite.

## 5 Conclusion

An extensible cardio-respiratory gating control unit that performs robustly during the delivery of a continual stream of rapidly switched and strong imaging gradients is described. The system enables prospective cardio-respiratory gating to operate without interruption to the maintenance of the steady state magnetization reliably so increasing image stability. In this report the techniques have been described with reference to bright blood 2D CINE imaging, but this gating scheme also enables the use of k-space ordered segmentation schemes such that cardio-respiratory synchronized imaging can be performed much faster than is possible with the retrospective gating techniques.

## Research data

Component details, computer programs and assembly instructions for the GCU; MRI pulse sequences and image reconstruction code, and the in vivo data presented in this report are available for download courtesy of the Bodleian Library at https://doi.org/10.5287/bodleian:pvRrYE7Kk and https://doi.org/10.5287/bodleian:xvRE1rmgm).
